# Exposure to nature versus relaxation during lunch breaks and recovery from work: development and design of an intervention study to improve workers’ health, well-being, work performance and creativity

**DOI:** 10.1186/1471-2458-14-488

**Published:** 2014-05-22

**Authors:** Jessica de Bloom, Ulla Kinnunen, Kalevi Korpela

**Affiliations:** 1 School of Social Sciences and Humanities, 33014 University of Tampere, Tampere, Finland

**Keywords:** Environmental psychology, Recovery from work, Park walking, Stress, Relaxation, Well-being

## Abstract

**Background:**

The objective of this research project is to understand and to improve workers’ recovery from work stress. Although recovery during lunch breaks is the most common within-workday break, it has received only minor research attention. Therefore, we will study whether lunch breaks including a relaxation session or exposure to nature have more favorable outcomes than usually spent lunch breaks concerning: a) recovery processes, b) health, c) well-being, d) job performance and e) creativity. We approach recovery by combining the theoretical frameworks of work and environmental psychology.

**Methods/Design:**

We conduct an intervention study in a sample of 268 knowledge-workers who engage in different lunch break activities for 15-minutes per day, two weeks in a row. We randomly assign participants to three experimental conditions: 1) exposure to nature, 2) relaxation and 3) control group (lunch break spent as usual). Online questionnaires before and after the intervention assess long term changes regarding recovery processes and the major outcome variables. Before, during and after the intervention, SMS and paper-pencil questionnaires measure the same constructs four times a day with fewer items. We also measure blood pressure and collect saliva samples to map cortisol excretion across the intervention period. A timed experimental task (i.e., the Alternative Uses Task) is used to examine differences in creativity between the three groups after the intervention period.

**Discussion:**

By combining the knowledge of work and environmental psychology about recovery and restorative experiences, by merging three recovery perspectives (settings, processes, and outcomes) and by using data triangulation, we produce valid results that broaden our view on mechanisms underlying recovery and enhance our understanding about their links to psychological, behavioural and physiological outcomes, resulting in a more comprehensive picture of work stress recovery in general.

**Trial registration:**

ClinicalTrials.gov Protocol Registration System NCT02124837. Registered 24 April 2014.

## Background

Employees exposed to stressful work conditions experience strain and suffer from poor well-being, which in turn has harmful effects on job performance and increases sickness absences. According to the European Working Conditions Survey, 30–40% of workers report mental health problems and stress-related disorders which are the biggest overall cause of early death in Europe [1,2].

It has been shown that unwinding from one’s job demands (i.e., recovery) is important for reducing the negative effects of work stress [3]. Recovery refers to the process during which an individual’s functioning returns to its pre-stressor level, and depleted resources are replenished [4]. In fact, in modern society, which is characterized by a hectic pace of life, efficiency and competitiveness in a global economy, it is likely that lack of recovery is a greater health problem than the absolute level of strain itself [5].

Recovery from work stress may constitute a protective mechanism that acts as a buffer in the work stress–strain relationship [3]. Accordingly, intervention studies which focus on improving the knowledge about and the skills to effectively recover from work stress are urgently needed [6]. Therefore, our intervention study is aimed at promoting recovery in workers. In doing so, we focus on lunch breaks as within-working day (internal) recovery.

Surprisingly, internal recovery has received much less attention than off-job (external) recovery [7], although most people spend about half of their day at their workplaces. Work breaks represent a period of time during which work-relevant tasks are neither required nor expected [8]. The study of within-day work breaks dates back to Mayo and the Hawthorne Studies in the 1930’s. However, since then the issue has not been examined with a specific focus on recovery from work stress and the underlying mechanisms (see [9], for a review). The few existing studies suggest that the types of activities people engage in during work breaks have implications for their well-being as well as their performance. For instance, feelings of recovery after work breaks were associated with more vigor and work-family facilitation before bedtime in a diary study [10]. Furthermore, Trougakos et al. [8] found that the type of activities service employees engaged in during their daily work breaks influenced their emotions and their affective displays in customer interactions. Restful and enjoyable activities (called respites) provided greater recovery than more effortful and not preferred activities (called chores). Respites may stop the depletion of regulatory resources and add affective resources. This conceptualization also matches another distinction made on the basis of the duty profile of activities: activities can be either resource consuming or resource providing [11].

In fact, it seems that rather than the specific nature of activities, it may be more important that the activities match individual preferences and needs (e.g., [12-14]). The mechanisms assumed to underlie the recovery phenomenon have recently been under study. Sonnentag and Fritz [15] have labelled these recovery experiences psychological detachment from work (disengaging oneself not only physically but also mentally from work; opposite to rumination), relaxation (a state of increased positive affect and low activation), mastery (challenging experiences and learning opportunities during off-job time), and control (ability to choose which activity, when and how to pursue during off-job time).

Psychological detachment and relaxation have their theoretical roots in the Effort-Recovery model [16]. When experiencing them, no further work-related demands act upon the psychobiological system. Mastery and control relate to the Conservation of Resources Theory [17] as they have the potential to rebuild depleted resources. Empirical evidence suggests that the four recovery experiences are helpful in recovering from work stress [18-20]. For instance, in a one-year longitudinal study [21] poor psychological detachment predicted job exhaustion one year later. In addition, feeling recovered after the weekend due to high levels of detachment, relaxation and mastery experiences has predicted higher levels of self-reported job performance during the following week [22].

In general, there is also a lot of evidence showing that relaxation is related to psychological (e.g., stress, anxiety), physiological (e.g., blood pressure, stress hormones, musculoskeletal pain syndromes, digestion) and organizational outcomes (e.g., productivity), although the latter two outcomes have been far less often examined [23-26]. However, most studies conducted so far are cross-sectional so that causality cannot be clearly established. Intervention studies with a quasi-experimental design are scarce in work- and organizational psychology, because they are expensive and difficult to implement in an organizational context [27].

In the current study, we do not only pay attention to the recovery process and its outcomes. Another important focus is the setting in which recovery takes place. The environment in which working people spend their lunch breaks may be essential for both their subjective experiences during the respite as well as the outcomes in terms of health, well-being and performance. This means that we approach recovery from work by combining the theoretical frameworks and empirical results of work and environmental psychology.

### Exposure to nature

Scientific evidence from the field of environmental psychology shows that restorative environments not only permit but also promote recovery from an inability to concentrate and from the elevated physiological arousal and negative emotions characteristic of acute stress and fatigue [28,29]. Exposure to nature can increase relaxation, improve well-being and job satisfaction [30,31]. A meta-analysis of 25 studies [31] comparing data before and after activities in natural environments showed significant decreases in negative feelings and increases in positive mood. Moreover, a pilot study combining work and environmental psychological aspects of recovery revealed a positive relationship between the time spent in interacting with nature and low need for recovery from work [32]. Spending time in a natural environment can decrease heart rate, muscle tension and skin conductance within a few minutes [33]. After approximately twenty minutes of exposure, blood pressure and salivary cortisol decreases and positive mood increases [34-36]. Even passively viewing urban parks or woodlands produces greater physiological changes toward relaxation, positive emotions, and faster recovery from attention-demanding cognitive performances than watching built environments without natural elements [29,35,37,38].

According to the Attention Restoration Theory [39], restoration of depleted resources unfolds in place-person interactions that involve preceding or co-occurring processes of a) fascination, i.e., effortless attention, b) psychological or geographical distance from one’s usual context, c) immersion in a coherent environment, and d) a good match between personal purposes, environmental supports and demands for action. These processes, also called processes of perceived restorativeness, typically appear more often in natural environments than in urban environments [40,41]. For instance several epidemiological studies indicate a positive relation between the amount of green space in the residential area and decreased morbidity and mortality rates [42-45].

An experimental study has focused on the impact of nature on well-being after simulated work. The student participants in this open-plan office laboratory worked for two hours in noisy setting, consisting of mobile tunes, telephone ringing and telephone conversations. After that, they took a 7-minute break in four different restorative conditions: 1) nature movie with sounds of streaming water, 2) river sounds only, 3) silence, 4) continuation of office noise [46]. The participants who saw the movie with sounds of water rated themselves as having more energy compared to the other three groups. No differences were found between the four conditions concerning stress hormones (i.e., salivary cortisol and urinary catecholamine) or cognitive measures after the break (i.e., solving logical problems, reading span & comprehension and serial recall).

Still, the current knowledge on the effect of exposure to nature during the workday is limited until now. Most studies were conducted in student samples and, accordingly have low external validity. Moreover, little is known about how long-lasting positive effects are and studies on working people have mainly focused on the determinants for exposure to nature rather than outcomes [47].

A recently published intervention study compared the effects of a 20-minute walk in nature and built environments during lunch breaks [48]. The 94 office workers of this study were instructed to walk during two lunch breaks per week for a period of eight weeks. There were no differences between the groups concerning autonomic nervous system activity (heart rate, heart rate variability, blood pressure, recovery from acute mental stress). Self-reported mental health increased in the nature walk group (compared to the built environment and the control group).

### Relaxation

Although there are some studies which have used relaxation and meditation techniques to improve employee well-being, these have often been combined with other techniques, such as time management [24] and to our knowledge only two studies have focused specifically on improving recovery processes during the work day in working people. One of these studies showed that it is possible to enhance recovery experiences (detachment, relaxation, control and to a lesser degree mastery) with a training program consisting of lectures as well as individual and group exercises, lasting a total of 9 hours in two weeks [49]. After the training, sleep quality increased, and stress as well as negative affect decreased. Another study, a controlled trial covering a period of 6 months, found that a relaxing break (consisting of 20 minutes progressive muscle relaxation in a silent room cabin) reduced post-lunchtime and afternoon emotional and motivational strain compared to a small-talk break group [50,51]. Moreover, there was a reduction in the immediate post-lunchtime and awakening cortisol states in the next morning. It is worth noticing, however, that this study focused on only 14 call center agents and was limited to strain outcomes (e.g., feeling nervous, relaxed, energetic, motivated).

### Outlook

To summarize, we approach recovery from three perspectives in this study: settings, processes, and outcomes [6]. Concerning settings, we focus on within-day recovery in the form of lunch breaks. Moreover, we take the environment in which recovery takes place into account. Concerning recovery processes, that is, mechanisms that underlie the recovery phenomenon, we examine especially psychological recovery processes (i.e. psychological detachment, relaxation, mastery and control; [15]), but also break (exposure to nature, relaxation) and leisure activities (physical, social, low-effort activities; [11,52]) including the environment in which they take place. Finally, our study incorporates different recovery outcomes (psychological, physiological and behavioral).

### Research questions and hypotheses

We conduct a field experiment focusing on enhancing the recovery potential of within-work breaks. The aim of this study is to examine the recovery value of two different break-time activities. These are a relaxation session and exposure to nature in the form of a park walk session. After having had lunch, randomly assigned participants either a) take part in a relaxation session, b) take a walk in a nearby park or c) spend their lunch break as usual (control group).

Our five research questions are: How does exposure to nature/relaxation during lunch breaks (compared to normal lunch breaks) affect:

1) recovery processes, i.e., restoration, relaxation, mental detachment, mastery and control during breaks

2) physiological health, i.e., health status, health complaints, blood pressure, cortisol excretion, health status, health complaints

3) (work-related) well-being, i.e., vigor, fatigue, stress, happiness, satisfaction, mood

4) job performance, i.e., task completion, ability to concentrate

5) creativity, i.e., fluency of ideas, cognitive flexibility, originality of ideas

Concerning the time frame, we focus on immediate effects (directly after the lunch break), short term effects (in the end of the work day), medium term effects (in the evening before going to bed) and long term effects. Concerning long-term effects, we distinguish between effects in the morning a couple of days later during the intervention period, effects during the first week after the intervention ended and three weeks after the intervention.

We also investigate whether certain variables during or after the work day, and person or job characteristics influence the potential link between exposure to nature/relaxation during lunch breaks and the outcome variables. More specifically, we focus on mediator/moderator variables during the work day (e.g., break characteristics, recovery processes, work demands), after work (e.g., free time characteristics) and in general (e.g., person characteristics, workaholism).

We hypothesize that exposure to nature and relaxation produce more favourable psychological recovery processes and short-term health, well-being and job performance than usually spent lunch breaks. We expect that improved psychological recovery processes may partially mediate the effects of the intervention on the major outcome variables.

We also hypothesize that poor recovery from job demands and deficient recovery processes may have negative long-term consequences. We expect that there may be certain combinations of psychological processes (e.g., dominated by high detachment and positive affect) which produce the best long-term outcomes. We do not have any specific hypotheses, but consider it possible that, for example, higher age and workaholism may intensify the negative effects of poor recovery from job demands on well-being, as it has been shown that recovery deteriorates along with ageing and that workaholism is a risk factor for well-being and health in general [53,54].

Concerning creativity, defined as “[…] the production of novel, useful ideas or problem solutions ([55], p.368), we hypothesize that exposure to nature positively affects creativity because Atchley et al. [56] demonstrated that spending time in natural surroundings is associated with increases in creativity, as measured with the Remote Associates Test. Regarding relaxation, most studies found that de-activating mood states produce less creative ideas than activating states [57]. Therefore, relaxation sessions may be less beneficial in terms of creative insights than actively spend lunch breaks.

## Methods/Design

The research plan has been approved by the Ethics Committee of the Tampere Region, Finland (Statement 10/2014). The data protection ombudsman has been notified in line with the Finnish Personal Data Act (523/1999), § 10 and § 14. The data collection will be divided into two phases in order to optimize material and personnel resources. The first phase takes place in spring (April-June) and the second phase in autumn (September-November). Both data collection phases cover a period of seven weeks (weeks 18–24 and weeks 35–41). These seven weeks include ten working days with each ten repeated measurements per measurement day (see Additional file 1: Table S1). Four measurements are scheduled in the morning (one blood pressure measurement, two saliva samples, one SMS questionnaire), one measurement after lunch (SMS questionnaire), two measurements at the end of the work day (one blood pressure measurement, one SMS questionnaire) and four measurements are planned before going to bed (one saliva sample, one blood pressure measurement, one SMS questionnaire, one paper-pencil questionnaire). The intervention covers a time span of two weeks. During these two weeks, participants are instructed to engage for 15 minutes during their lunch break on working days (altogether 10 days) in one of the activities they are randomly assigned to within each company: 1) exposure to nature (i.e., park walking), 2) relaxation exercises, 3) usual break activities (control group).

Two weeks before the intervention, the participants also fill in an online questionnaire with questions regarding demographics (e.g., age, marital status, education), basic job information (e.g., weekly work hours), break habits during the work day (frequency, duration and location of breaks, activities during breaks), well-being (e.g., work engagement, burnout) and health (e.g., physical diseases, Body Mass Index). Moreover, baseline levels of the major outcome variables are assessed in depth at this point in time. The online questionnaire can be approached by clicking on a link in an email sent to the participants. At the end of the research period (week 24 or 41 respectively), a second online questionnaire is used to assess break habits and scores on the major outcome variables in detail for the second and last time.

One week before the intervention, during the intervention period, as well as in the first and the third week after the intervention, measurements are scheduled on Tuesdays and Thursdays. On these days, three different types of measurements take place 1) SMS questionnaires, 2) paper-pencil questionnaires, 3) blood pressure measurements (self-administered) and 4) saliva sampling (self-administered). SMS-questionnaires are sent to the participants’ cell phone four times per day: early in the morning (7.00), after lunch (11.30), at the end of the work day (16.00) and in the evening before going to bed (21.00). The SMS questionnaires are programmed into a digital system developed by a team of researchers at the University of Applied Sciences in Jyväskylä called eGRoup coach. When the system has been developed, special attention has been paid to data protection and privacy and it has been successfully applied in diary studies before [58,59]. Every participant is assigned a personal code which he/she has to type in before answering the questions. This code also makes sure that the answers are anonymized (e.g., important if the phone is lost or stolen) and belong to the person in question. The questionnaires are very short and it takes no longer than 3 minutes to reply to the questionnaires by replying via SMS. The costs for the replies are covered by the research project. At the end of each measurement day, participants also reply to a number of questions presented to them in a paper-pencil booklet where they also note down their blood pressure values. The participants are requested to measure their blood pressure three times per day: early in the morning, 30–60 minutes before the end of their work day and in the evening before going to bed. Participants are instructed to collect saliva three times per day: right after awakening, 30 minutes after awakening and before going to bed.

We take several steps to secure commitment from participants and reduce non-response as suggested by Newman [60]. For example, each participant gets a personal time schedule of his/her measurement occasions and each measurement day is preceded by a reminder during the preceding evening. During the intervention weeks, the participants in the two intervention groups receive a reminder SMS instructing them to engage in park walking/relaxation during their next lunch break. On a daily basis, we will check whether participants replied to the daily SMS questionnaire. In case of two missed measurements, we will call the participants to ask why they do not reply to the SMS messages (to detect technical errors) and emphasize that adherence to the research protocol is essential for the success of this study.

### Study population and recruitment

We recruit workers with knowledge-intensive and emotionally demanding jobs as it is probable that work stress recovery problems concern especially these workers. Workers with knowledge-intensive jobs often have so-called boundaryless jobs in which flexibility in terms of time, space, and organization of work is typical [61]. This shifts the responsibility for the limits of work to the workers themselves. Knowledge work is often also sedentary, which seems to increase health risks regarding higher BMI and diabetes [62]. Emotional work is often done in the service sector and demands a high level of positive affective displays. This challenges workers’ behavior regulation as they have to present the appropriate emotional displays on demand. This kind of work can be quite taxing, and it has been related to impaired well-being [63].

**Figure 1:**
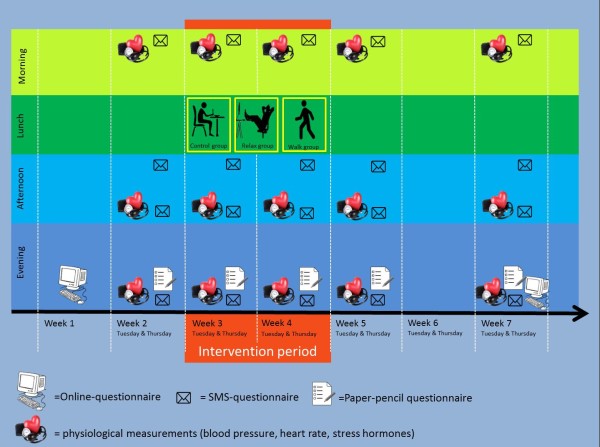
Overview research design for participants in spring 2014; screenshot of power point slide used during recruitment stage (sent by email).

The participants are mainly recruited with the help of a company, Tampereen Työterveys Ry., which supplies occupational health service to 848 client organizations. The company will send an email to some of their client organizations, introducing our study. A few days after receiving the email, we will contact the organizations by telephone and ask them if they want to take part in our study. If they agree, they may provide us with the email addresses of their employees or forward our recruitment email to their workers (see Figure 1). This email includes a link to a short online registration questionnaire (asking name, address, birth date, name of the employer and checking the exclusion criteria). Exclusion criteria for this study are a) shift work or highly irregular working hours, b) absence of a park nearby and c) serious illness or allergies that prevent workers from going outside for a walk. A company can take part in the study if there are minimally six employees willing to join. Participants from companies with fewer than six volunteers will be invited to fill in the two online questionnaires before and after the intervention period. The companies who participate in our study will be randomly assigned to the first or the second phase of the study (spring or autumn). Within each company, the participants who agreed to take part will be randomly divided into three groups (exposure to nature, relaxation, control group). The spring intervention study includes 112 persons from seven different organizations and 51 additional persons will fill in the online pre- and post-questionnaires only to control for testing effects. In the autumn, another 105 persons will participate in the study, resulting in an overall sample size of 268 persons.

In the month before the study starts, participants are invited to attend a training which lasts approximately two hours. The training is organized on site for maximally ten participants at a time. Trainers are work- and organizational psychologists (or students in an advanced stage of their studies) who have been briefed and thoroughly trained by the researchers and who attended an intensive three-hour relaxation training session. During the training, the exact procedure of the study is explained to the participants and they have the possibility to ask questions about the data collection procedure. Moreover, participants get detailed written and verbal instructions for each type of measurement (online questionnaires, SMS questionnaires, paper-pencil questionnaires, saliva sampling, blood pressure measurements) and the trainer makes sure that each participant practices each type of measurement at least once during the training. In this way, the participants become familiar with the measurement devices used in this study. An important part of the training concerns the signing of the informed consent form to make sure that participants understand that participation is voluntary and that they can withdraw from the study at each point in time.

After the data collection is finished, every participant receives information on his individual values of blood pressure and well-being compared to the average levels of the whole group. We will also have a lottery in which it is possible to win three prizes: one travel check for 200€ and two checks for 100€. We communicate that more completed measurements will increase the individual chance on winning.

### Experimental conditions

#### Exposure to nature

After the training session for all participants, the participants who are assigned to the nature exposure group are instructed to walk a predetermined route in the park at a slow, low-intensity pace, to pay attention to their surroundings and to avoid discussion for 15 minutes during their lunch break. The trainers walk the route one time with the group during the training and participants are handed out maps with the route marked on it. Before and after the park walk, participants indicate their level of relaxation on a paper sheet. They can walk either alone or in a group.

#### Relaxation

The relaxation session during the lunch break is based on applied relaxation, which is the most studied and applied stress reduction method [24]. In the relaxation session, the focus is on deep breathing, progressive muscle relaxation and acceptance exercises based on mindfulness meditation (e.g., [64,65]). The exercises are especially designed to make use of the autonomic nervous system potential and have been successfully used in clinical settings [66]. The participants in the relaxation group are trained on this method for 1.5 hours before the start of the intervention period by a trained psychologist or a trained psychology student. They also learn how to test their level of physical relaxation. The relaxation session lasts 15 minutes and it needs only a chair to be performed. Before and after each relaxation exercise, participants indicate their level of relaxation on a paper sheet.

### Measures

#### Manipulation check

Before and after the lunch break intervention, participants in the nature/relaxation groups indicate their level of relaxation on a scale from 0 to 100 on a sheet of paper. The scale is anchored: 0 = extremely relaxed, such as sitting on the couch after exercising and having a sauna bath, 50 = normal level of relaxation/tension, 100 = extremely tense, such as before a major, stressful life event or an important verbal examination in public. These evaluations are marked in the booklet giving also written instructions for each exercise.

### Recovery processes

#### Restoration during lunch breaks

We measure recovery experiences with an online questionnaire before and after the intervention consisting of 9 items. The items focus on feelings and thoughts during lunch breaks related to relaxation, energy levels and mental disengagement from work. Example items are: “I worry about my work during my lunch break” and “I feel restored and relaxed after my lunch break”. The items are inspired by different existing scales that have been successfully applied in recovery studies such as the Recovery Experience Questionnaire [15], the Subjective Vitality Scale [67,68], the Energy at Work Scale [69] and the Restoration Outcome Scale [70]. Response scales range from 1 (very seldom or never) to 5 (very often or always).

Using paper-pencil questionnaires at the end of the day, we measure recovery experiences on day-level as follows:

#### Relaxation

Relaxation during the lunch break and in the evening is assessed by one item from the Recovery Experience Questionnaire [15]. Participants reply to statements such as: “During my lunch break, I took time for leisure”. In the evening, relaxation is measured by the item: “I kicked back & relaxed”. The Likert-response scale ranges from 1 (strongly disagree to) to 5 (strongly agree).

#### Psychological disengagement

Disengagement from work is measured daily by two items: “During my lunch break, I distanced myself from my work” and “During time after work, I distanced myself from my work”. The response scale ranges from 1 (strongly disagree) to 5 (strongly agree).

#### Positive work reflection

Positive thoughts about one’s work during recovery episodes are assessed by two items, adapted for the time of the day: “During my lunch break/during time after work, I reflected on the positive aspects of my work”. Participants can respond on a scale from 1 (strongly disagree) to 5 (strongly agree).

### Health

#### Health status

One item assesses the health status of the participants in the online questionnaires before and after the intervention: “How would you rate your general health status?”. The Likert response scale ranges from 1 (very unhealthy) to 10 (very healthy).

#### Health complaints

Physical complaints are measured by a shortened version of the Physical Symptoms Inventory [71] in the online questionnaire before and after the intervention. Participants are asked whether they experienced certain symptoms during the past month. Listed are the most frequently occurring symptoms such as headache, gastrointestinal problems or dizziness. The last question leaves an open space to report “other symptoms”. Respondents report the frequency of the symptoms: 1 (almost) never, 2) about once per month, 3) a few times per month, 4) about once per week, 5) a few times per week, 6) (almost) every day. If a participant experiences any of the symptoms, he/she is also asked to indicated if/how the complaints limited engagement in usual activities ranging from 1 (not at all) to 5 (completely; could not engage in my usual daily activities). In the paper-pencil questionnaires, participants report their degree of agreement (i.e., 1 = strongly disagree to 5 = strongly agree) with the following statement: “Today at work, I suffered from physical complaints such as headaches, an upset stomach, neck or back pain”.

### Well-being

#### Work engagement

Vigour and dedication are assessed with six items from the cross-nationally validated Utrecht Work Engagement Scale [72-74] via an online questionnaire before and after the intervention. The participants can answer on a scale ranging from 0 (never) to 6 (always/every day). On day-level with paper-pencil questionnaires, participants indicate how they experienced the thought of starting work (“This morning, I felt like going to work”). Answering scales run from 1 (strongly disagree) to 5 (strongly agree).

#### Fatigue

In the online questionnaire before and after the intervention, participants are asked how often they felt tired during the day within the last month. Answers can range from 1 (very seldom or never) to 5 (very often or always). On day-level with the SMS questions, fatigue is assessed four times a day by a one-item questionnaire developed and validated by Van Hooff et al. [75]. Participants report their level of agreement on a scale from 1 (strongly disagree) to 5 (strongly agree).

#### Burnout

Exhaustion and professional efficacy are measured with eleven items from the Finnish translation of the Maslach Burnout Inventory [76,77] before and after the intervention with an online questionnaire. Answers can range from 0 (never) to 6 (always/every day). On day-level, with paper-pencil questionnaires, exhaustion and personal efficacy are measured with one item each. The scale is adapted to the daily context and, accordingly, ranges from 1 (strongly disagree) to 5 (strongly agree).

#### Stress

Stress symptoms are assessed on day-level with a one-item scale inspired by Elo, Leppänen and Jahkola [78]. At the end of the work day, participants indicate their level of agreement (i.e., 1 = strongly disagree to 5 = strongly agree) to the following statement sent to them by SMS: “Right now, at the end of my work day, I feel stressed and tense”.

#### General well-being

This construct, measured online before and after the intervention, includes one item measuring happiness: “How happy do you feel in general?” with a Likert-scale from 1 (very unhappy) to 10 (very happy) and one item measuring satisfaction: “How satisfied do you generally feel about your life?” with a scale from 1 (very dissatisfied) to 10 (very satisfied). Moreover, happiness is assessed four times per day with the item: “I feel happy” with answers ranging from 1 (strongly disagree) to 5 (strongly agree).

#### Sleep quality

The quality of sleep is measured in the online questionnaires before and after the intervention as well as with paper-pencil questionnaires on day-level. The items are based on the Sleep Quality Index developed by Kecklund and Akerstedt [79]. The time span that the questionnaire refers to is shorted from six month to the last month in the online questionnaires. In these questionnaires, four items assess difficulties falling asleep, awakenings during the night, premature awakening and the degree of feeling refreshed in the morning. Answers can range from 1 (very seldom or never; less than once a month) to 5 (very often or always; daily/nearly daily). On day-level, sleep quality is assessed with one item: “How did you sleep last night?”. Response scale ranges from 1 (very poorly) to 5 (very well).

### Job performance

#### Task completion

The degree to which employees finish their daily work tasks is measured once per day with the following item: “Today, I completed all the tasks I wanted to complete” [80]. Answers can range from 1 (strongly disagree) to 5 (strongly agree).

#### Concentration

The ability to concentrate on daily work tasks is measured with one SMS-item after lunch and at the end of the work day. The item is inspired by the Questionnaire on the Experience and Evaluation of Work [81] and reads: “My ability to concentrate is…”. Workers can choose between five response options from 1 (very poor) to 5 (very good).

### Creativity

We use the Alternative Uses Task, a widely used, well-validated, reliable measure of creativity which measures the ability to produce a broad range of associations to a given stimulus [82,83]. In this divergent thinking task, people generate ideas in response to written prompts. Respondents are asked to write down all uses they can imagine for a brick. The task is included in the online questionnaire sent to the participants at the end of the research period. They have two minutes time to type their answers in a pre-defined field. After these two minutes, the participants are instructed to select their two most creative answers from all the answers they have given. After the data collection, the answers of all respondents are scored on fluency, cognitive flexibility and on originality by three trained raters independently.

#### Fluency

Fluency describes the number of ideas generated for the task (i.e., the number of uses a person can invent for a brick).

#### Cognitive flexibility

Cognitive flexibility is considered the mental core of creativity [84] and it describes the ability to break common patterns of thought, to overcome functional fixedness and to avoid a reliance on conventional ideas or solutions [82]. Flexibility becomes apparent by the number of conceptual categories an individual’s response can be assigned to. The greater the range of ideas, the more flexible a person is considered to be. To calculate mean scores of flexibility, three raters count the total number of different categories that a respondent’s idea belongs to. For example, if a respondent comes up with ideas of building a house and building a shed, both ideas fall into one category (i.e., building) and the resulting score is one. If a respondent suggests building a house and using the brick as paperweight, he/she would receive two points, because the ideas cover two different categories (i.e., building and using the brick as a weight). Before rating, the three raters carefully read all answers and agree on the categories the ideas could possibly belong to.

#### Originality

Originality represents the degree to which an idea is collectively considered uncommon, remote and clever. To rate the level of originality of a respondent’s answer, three trained raters independently score the originality of every generated idea of each respondent. Raters score each single idea on a five-point rating scale from 1 (not at all creative) to five (extremely creative). The raters are provided with a written instruction, telling them to keep in mind the following three aspects of creativity: 1) Creative ideas are uncommon and occur infrequently in a sample. Unique responses tend to be more creative responses. 2) Creative ideas are remote. They link everyday objects and concepts and stray from obvious ideas. For example, creative uses for a brick are far from common, everyday normal uses for a brick such as building a house. 3) Creative ideas are clever. They strike people as insightful, ironic, humorous, fitting, or smart. For each idea, the resulting three individual ratings are averaged. To calculate an overall originality score for each respondent, the respondents’ ratings for each single idea are summed and divided by the total number of his/her responses to control for verbal fluency or typing speed [83]. Moreover, a sum score is calculated for the two ideas that the participants designated as their most creative ideas. This Top 2 rating seems to be one of the best, most valid indicators for creative thinking [83].

### Potential moderators, mediators and control variables

#### Recovery processes

Recovery processes, such as control (“I determined for myself how to spend my time”) and the other recovery experiences described above are also investigated as moderator or mediator variables in the relation between work load, strain, health and well-being. The manipulation check (i.e., whether workers were able to relax during their lunch break and whether they adhered to our walking instructions) is also taken into account.

### Break characteristics

#### Enjoyment of activity

The participants of our study can indicate the degree to which they enjoyed their lunch break ranging from 1 (strongly disagree) to 5 (strongly agree) on the statement “I enjoyed my lunch break”.

#### Company

We inquire if participants have spent their lunch break alone or with others.

#### Break environment

We ask participants if they have spent their lunch break inside or outside their office.

### Work demands and resources

#### Workload

Before and after the intervention, work load is measured with three items from the Quantitative Work Load Inventory, developed and validated by Spector and Jex [71]. An example item is: “How often does your job require you to work under time pressure?”. Participants can respond on a 5-point scale (1 = very seldom or never, 5 = very often or always). On day-level, work load is measured by the SMS-statement: “My work demands were high today” on a scale ranging from 1 (strongly disagree) to 5 (strongly agree).

The following job demands and resources are assessed once before the intervention in the online questionnaire:

#### Cognitive demands

Cognitive job demands are assessed with three items. The questions are based on the Copenhagen Psychosocial Questionnaire [85] and the DISC Questionnaire [86]. One of the items reads: “How often does your work require you to make complex decisions?”. The response scale is identical with the scale described above.

#### Emotional demands

The emotional demands of the participants’ jobs are assessed by three items from the Copenhagen Psychosocial Questionnaire [85]. An example item is: “My work is emotionally demanding”. The response scale also ranges from 1 to 5.

#### Work variety

The variety a participant’s job offers him/her is measured with three items from the Questionnaire on the Experience and Evaluation of Work [81]. An exemplar question is: “Do you have enough variety in your work?”. The response scale is the same as above.

#### Autonomy

Five items assess autonomy in a participants’ job. Four of these items stem from the General Nordic Questionnaire for Psychological and Social Factors at Work [87] and one item is derived from the Psycones Questionnaire [88]. The answers are recorded on the 5-point frequency scale described above.

#### Social support at work

Support from colleagues as well as support from supervisor are assessed with three items from the General Nordic Questionnaire for Psychological and Social Factors at Work [87] and the Questionnaire on the Experience and Evaluation of Work [81]. An example item is: “I get on well with my nearest supervisor”. The response scale is the same as described above.

#### Work environment

Participants are asked several questions about the type of office they work in. The response categories are: 1 = Private office for one person, 2 = Shared office space with a personal desk 3 = Shared office without a personal desk 4 = Permanent classroom/teaching room, 5 = Changing workspace, class- or teaching room and 6 = Other. If the participants share their work space with others, we inquire into the number of colleagues and clients that the space is shared with. The third question concerning the work environment concerns the window view: “Do you have a window, a glass door or glass wall at your room/working station?”. Participants can choose between the following answers: 1 = No, 2 = Yes, it is to the inside of the building, 3 = Yes, it is to the outside of the building with mainly an urban view (for example a building or street) 4 = Yes, it is to the outside of the building with mainly a natural view (for example a lake, field or park).

### Free time characteristics

#### Type of free time activities

On day-level, we ask participants how many hours they have spent on the following activities after finishing work: work-related activities, home chores, physical activities, physical activities in nature and social activities.

### Person characteristics

The following person characteristics are assessed with an online questionnaire two weeks prior to the intervention:

#### Workaholism

Working compulsively and working excessively are assessed with three items from the Brief Workaholism Scale, developed by Schaufeli, Van Wijhe, Peeters and Taris [89] and validated by del Libano, Llorens, Salanova and Schaufeli [90].

#### Mindfulness

The two key components of mindfulness, “acting with awareness” and “acceptance” are measured by a scale composed of six items in total. Three of the items stem from the Mindfulness Attention Awareness Scale [91] and three from the Kentucky Inventory of Mindfulness skills [92]. The items are chosen based on the highest factor loadings reported in American, Chinese and Swedish samples [93-95].

#### Background variables

We ask the respondents to report their age, gender, formal education, family status, tenure and if they have a supervisory position.

### Physiological measures

We collect saliva samples and measure blood pressure. Both procedures are minimally invasive for participants. To protect subjects’ privacy, the booklets in which they write down the values of their blood pressure and the collection times only contain an identifier and no name. We use text-messages to remind the participants about the measurements. Written instructions on how to collect the data are handed out to the participants and discussed during the training session.

#### Saliva sampling

Participants collect saliva samples with the help of Salivette swabs. During a training session before the intervention, participants are instructed how to use the swabs. Via SMS, every participant receives a reminder to collect saliva three times per day: right after awakening, 30 minutes after awakening, and before going to bed (see Additional file 1: Table S1). These three measurements are minimally required in order to capture the diurnal course of biomarker excretion. The swabs are labeled by a certain number and distributed in separate plastic bags labeled with the week of the measurement. The participants are instructed to refrain from eating, drinking, smoking, exercising and brushing teeth within 30 minutes before collecting saliva to prevent confounded samples. The participants are also instructed to mark if they complied with these guidelines in the paper booklet. We ask the respondents to rinse their mouth before collecting saliva and we instruct them to place the swab between the cheek and the lower teeth for one minute, as location and movement in the mouth may influence the concentration of biomarkers [96,97]. After sampling, the participants are asked to keep the samples in their fridge until the researchers collect them at their work place. The samples are analyzed for cortisol by the physiological laboratory of the Finnish Institute of Occupational Health. Cortisol represents a valid measure for hypothalamic-pituitary-adrenal (HPA) axis activity [98,99]. In statistical analyses, special attention is paid to possible confounders (e.g., age, gender, smoking, alcohol consumption, medication, sleep, exercise).

#### Blood pressure measurements

High blood pressure has been shown to be the most important risk factor of cardiovascular disease, which is the most common group of diseases in mortality and morbidity. Blood pressure reflects sympathetic and parasympathetic activity and balance of the autonomic nervous system and is elevated during stress [100,101]. The participants measure their blood pressure three times per day: in the morning, at the end of their work day and before going to bed (see Additional file 1: Table S1). The participants are instructed not to eat or drink, smoke or exercise 30 minutes prior to measuring their blood pressure. Blood pressure is assessed with a fully automatic blood pressure monitor which uses the oscillometric method for detecting the blood’s movement through the brachial artery and converting it into a digital reading (OMRON, model M2, validated and recommended by the British Hypertension Society). We instruct the participants to conduct the measurements in a sitting position in a quiet place and to avoid talking during the measurement. We also ask them to take two separate measurements per occasion to get a more reliable indicator of their current blood pressure level. Odd measure out of range (e.g., due to a misapplied arm cuff) are deleted from the analyses. In statistical analyses, special attention is paid to possible confounders (e.g., age, gender, smoking, alcohol consumption, medication, sleep, exercise).

### Statistical analyses

#### Sample size calculation

We calculated that an effect size of .25 (medium) with alpha = .05, and power = .95 would require 84 respondents per group in one-way ANOVA (three groups) [102]. If non-adherence occurs, 53 participants per group suffice with a power level of .80 [102,103]. In field experiments, smaller samples have typically been used (e.g., [50]). Note that in ANOVA, to assess within-between interaction effects with 10 repeated measurements with 0.3 correlation between them and three groups, a total sample size of 36 is sufficient to produce an effect size of .25 (medium) with alpha = .05, and power = .95 [103].

#### Basic analyses

Analyses are performed with SPSS 20.0 [104] and MPlus [105]. A two-tailed significance level of < .05 is considered statistically significant. The characteristics of the sample are analyzed using descriptive statistics. Independent samples t-tests or chi-square tests are applied to test whether randomization was successful or whether there are any systematic differences between the three groups of workers. Preliminary analyses include calculating descriptive statistics (i.e., means, standard deviations and range of scores), factor analyses for all the scales used, computing internal reliabilities (Cronbachʼs α) and bivariate correlations (Pearsonʼs *r*) of all variables in the study.

#### Effect evaluation

Repeated measures ANOVA’s are applied to test the change in outcomes within subjects across the ten repeated measurements before, during and after the intervention (i.e., within-subjects main effect of exposure to nature/relaxation during lunch breaks). To test how the different types of break activities affected the change in outcomes across time, the three experimental groups, 1) relaxation, 2) exposure to nature, 3) control), are entered as a between subjects factor (i.e., between subjects main effect for break activity type). Partial eta-squared (η_p_^2^) is reported as an effect size. We use a sequential Bonferroni procedure to control for Type I error across the analyses. In this procedure, tests are placed in ascending order of significance within a family of tests. The smallest probability is then multiplied by the number of tests in the family. The second probability is then multiplied by the remaining number of tests, and so on. Tests are judged to be significant if the product is smaller than .05.

Concerning cortisol, we calculate the total daily production by using the area-under-curve with respect to ground. Moreover, we will calculate the slope of the diurnal profile. We also control the physiological analyses for certain variables. On day-level, we may control for: exercising, eating or drinking within the last 30 minutes (stress hormones, blood pressure), wake up time, sleep time, acute illness or pain, smoking, age and brushing teeth. On a general level, we may control for age, gender, use of oral contraceptives, pregnancy, body mass index, too high or too low blood pressure, endocrine or hormonal diseases and psychological diseases. We may also decide to exclude the physiological data of certain persons from the analyses if they, for example, not adhered to our instructions or suffer from a disease that may impact their biomarker levels.

#### Moderator and mediator analyses

To test moderation and mediation hypotheses, we check the variables for multicollinearity first, because it may attenuate or suppress the effects of individual predictors on the outcome variables. Our criterion for acceptable multicollinearity among mediators/moderators is less than 10% of common variance (see [106]). We apply nonparametric bootstrapping (with 5,000 bootstrap resamples) utilizing a SPSS macro presented by Preacher and Hayes [107]. This method does not impose the assumption of normality of the sampling distribution, provides high statistical power and reduces the likelihood of Type I error [108,109]. If covariates vary in time, we will use structural equation modeling (that is, latent growth modeling) to model changes and inter-individual variability across time.

#### Process evaluation

A process evaluation is carried out to understand which factors influenced the effectiveness of the intervention. Therefore, at the end of the study, all participants are invited to comment on the research procedure by email. Moreover, a subsample of ten randomly selected participants is called and interviewed by phone about their experiences.

## Discussion

This article describes the development and the design of an intervention study which aims to improve workers’ recovery during lunch breaks by either exposure to nature or carrying out relaxation exercises. The aim of this study is to examine work stress recovery from the process perspective, paying special attention to the underlying recovery mechanisms that promote or hamper physical and psychological restoration.

### Strengths and limitations

The findings of this study are subject to at least five limitations. Firstly, the participants are aware of the purpose of the study which may lead to placebo effects. However, as we combine different outcome measures and tools (e.g., online questionnaires, SMS questionnaires, experimental tasks, physiological measures), we assume that placebo effects on the subjective outcome measures would become apparent in the more “objective” measures such as physiological measurements and experimental tasks. Moreover, we also include a group of persons who only fill in the pre- and post-intervention questionnaires. By comparing the outcomes of this group to the control group’s outcomes, we can establish internal validity and rule out testing and/or placebo effects.

Secondly, the study is subject to several other threats to internal validity such as history and maturation. The season of the year (spring versus autumn) may also influence health and well-being levels of the participants. So, before combining the participants from the two phases of data collection, we will test whether any systematic differences exist. Thirdly, repeatedly measuring the same constructs in the participants may lead to testing bias. Especially replying to the same questions a couple of times per day may become boring. Therefore, we kept the number of items which measure the underlying constructs to a minimum. Furthermore, the order of the questions will be varied between the measurement days. Fourthly, diffusion may occur. This means, the employees in the control group may feel motivated to join their colleagues during their walking or relaxation session at lunch time. In order to prevent this, we will explain why it is important for the control group to refrain from engaging in the same activities as the intervention group or changing their usual habits for the duration of the intervention study. Moreover, we ask the control group how they spent their lunch break so that we could exclude persons who changed their break routines. Fifthly, we have to take into account that workers will not adhere to the instructions, forget to reply to some questionnaires or to take certain measurements. Some workers may drop out completely. In order to deal with these problems, we 1) stress the importance of adherence to the research protocol, 2) stress the problem of “cheating” (i.e., taking physiological measurements at a wrong point in time), 3) intervene when certain employees forget repeatedly to fill in questionnaires, 4) ask participants whether they adhered to the guidelines for the physiological measurements at each measurement occasion, 5) test whether missing data are distributed completely at random (using Little’s MCAR test). If the data is not missing at random, we will impute the missing data.

The strengths of our study boil down to four main assets. Firstly, interventions in working samples are scarce and even less common are studies focusing on recovery from work during the work day. Therefore, our study contributes to our understanding of recovery processes, while it also has direct practical implications for working people. If the strategies we apply indeed improve recovery from work during lunch breaks, they constitute an easy and quick way to enhance workers’ health, well-being, performance and creativity. Our study also helps to establish causal links between the variables under investigation. Secondly, most studies in the field of creativity lack external validity, because they are carried out in student samples [110]. Our study fills this gap by applying an experimental task to measure creativity in a working population in a field setting. Thirdly, combining different biomarkers of stress (i.e., cardiovascular stress responses and salivary cortisol) contributes to a better understanding of the interaction between sympathetic system and HPA axis activity. In addition, the combination with other, more subjective, indicators of health and well-being, performance and creativity will provide new insights into the interplay between body and mind. Fourthly, in combining theories and instruments from two research fields, environmental and work and organizational psychology, that have long been separated, we hope to promote further cross-fertilizations between research areas.

## Conclusions

By combining the knowledge of work and environmental psychology about recovery and restorative experiences (covering especially emotional and cognitive processes) and by merging three recovery perspectives (settings, processes, and outcomes) into one single study, we broaden the view on mechanisms underlying recovery and enhance our understanding about their links to psychological, behavioural and physiological outcomes, resulting in a comprehensive picture of work stress recovery in general.

## Competing interests

The authors declare that they have no competing interests.

## Authors’ contributions

JdB drafted the manuscript and participated in its design and coordination. UK conceived of the study, and participated in its design and coordination. KK conceived of the study, participated in designing the study and performed several statistical analyses. UK and KK both revised the manuscript. All authors read and approved the final manuscript.

## Pre-publication history

The pre-publication history for this paper can be accessed here:

http://www.biomedcentral.com/1471-2458/14/488/prepub

## Supplementary Material

Additional file 1: Table S1 Research design and time investment from the participants’ point of view. Click here for file
