# Supplementary figures and images for: Exposure to nature versus relaxation during lunch breaks and recovery from work: development and design of an intervention study to improve workers’ health, well-being, work performance and creativity
Source: BMC Public Health. 2014 May 22;14:488. doi: 10.1186/1471-2458-14-488 (PMC4039544; doi:10.1186/1471-2458-14-488)

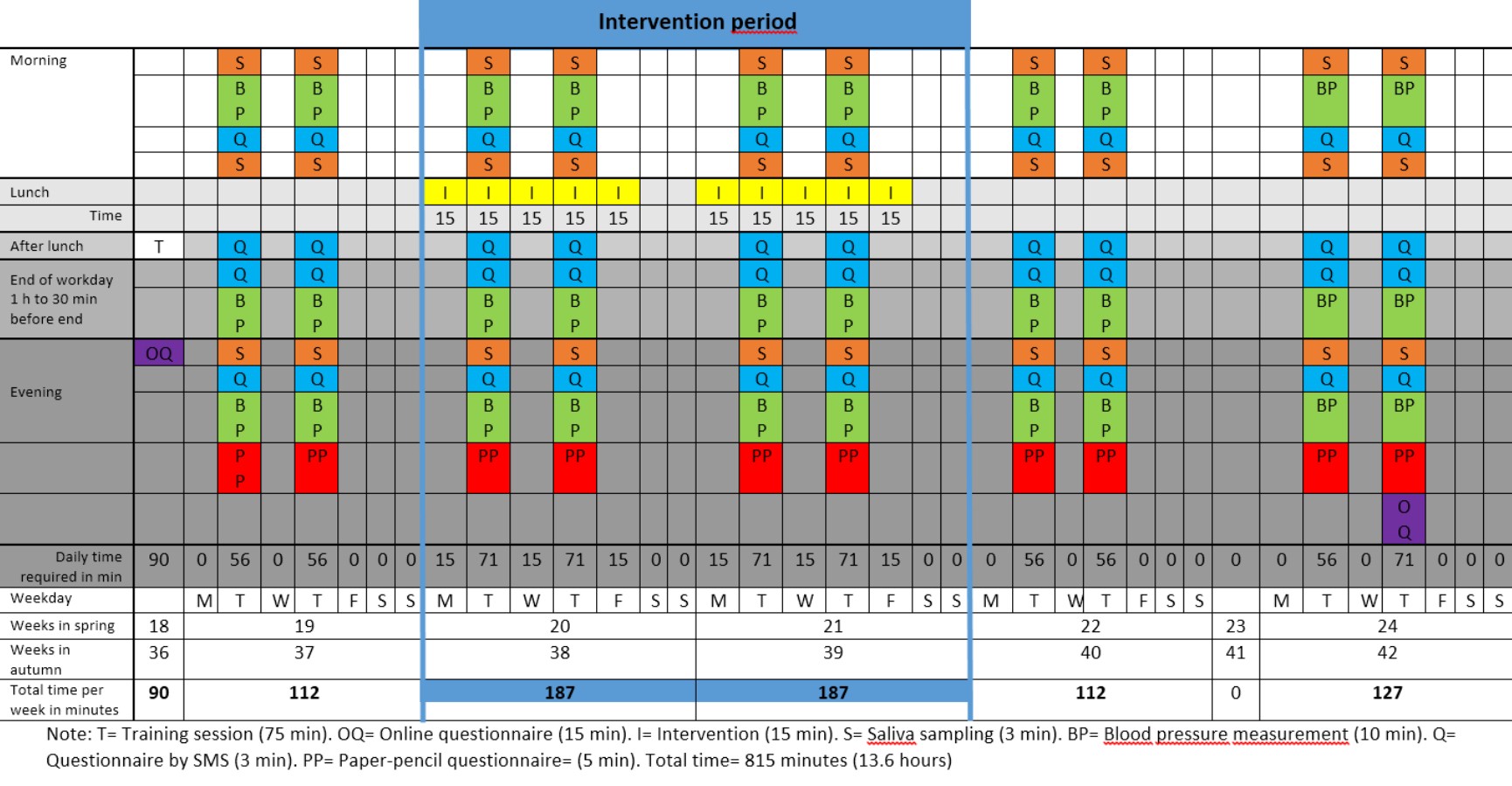

Supplement: Additional file 1: Table S1 — Research design and time investment from the participants’ point of view. [file 1471-2458-14-488-S1.jpg]
